# Enhancing aesthetic appreciation by priming canvases with actions that match the artist's painting style

**DOI:** 10.3389/fnhum.2014.00391

**Published:** 2014-06-03

**Authors:** Luca F. Ticini, Laura Rachman, Jerome Pelletier, Stephanie Dubal

**Affiliations:** ^1^Institut du Cerveau et de la Moelle Épinière, ICM, Social and Affective Neuroscience (SAN) LaboratoryParis, France; ^2^Sorbonne Universités, UPMC Univ Paris 06, UMR S 1127Paris, France; ^3^Inserm, U 1127Paris, France; ^4^CNRS, UMR 7225Paris, France; ^5^Institut Jean Nicod, CNRS-EHESS-ENS UMR 8129Paris, France; ^6^The Italian Society for Neuroaesthetics ‘Semir Zeki’Trieste, Italy

**Keywords:** action, priming, simulation, aesthetic appreciation, art, mirror neurons

## Abstract

The creation of an artwork requires motor activity. To what extent is art appreciation divorced from that activity and to what extent is it linked to it? That is the question which we set out to answer. We presented participants with pointillist-style paintings featuring discernible brushstrokes and asked them to rate their liking of each canvas when it was preceded by images priming a motor act either compatible or incompatible with the simulation of the artist's movements. We show that action priming, when congruent with the artist's painting style, enhanced aesthetic preference. These results support the hypothesis that involuntary covert painting simulation contributes to aesthetic appreciation during passive observation of artwork.

## Introduction

Perceptual, cognitive, and affective evaluations contributes to the aesthetic experience of a work of art (Cela-Conde et al., 2004; Kawabata and Zeki, 2004; Vartanian and Goel, 2004). Although much research has focused on reward-related brain regions involved in artistic preference (above all the oribitofrontal cortex; Jacobsen et al., 2006; Ishizu and Zeki, 2011, 2013; see also Ticini and Omigie, 2013), the role of other brain structures has remained thus far poorly explored. Here, we investigate the contribution of motor areas to aesthetic experience, a topic of very wide interest (Freedberg and Gallese, 2007). Several neuroimaging experiments have shown that the perception of artworks elicits motor activity in the observers' brain without fully clarifying its role in aesthetic experience (Kawabata and Zeki, 2004; Cela-Conde et al., 2009; Cross et al., 2011; Ishizu and Zeki, 2011, 2013; Cross and Ticini, 2012; Umiltà et al., 2012; Sbriscia-Fioretti et al., 2013). Indeed, on the one hand, motor activity may simply be triggered by a covert approach or avoidance response related to the emotional nature of the artwork, as it has been shown for other types of stimuli (Hajcak et al., 2007). On the other, some have hypothesized that it may represent the covert and involuntary simulation of the artist's gestures when viewing a work of art, signs of which may be present on the canvas in the form of brushstrokes (Freedberg and Gallese, 2007). Whether the latter interpretation is correct and whether motor activity contributes to the aesthetic experience at all, is still unclear.

We recorded the preference of naïve individuals for 90 high quality reproductions of pointillist-style paintings presented under conditions specifically designed either to be compatible or not with the actions required to produce them (as established in associative training conducted beforehand, see Materials and Methods). Each painting was preceded by a supraliminal priming consisting of a static image depicting a hand either holding a paintbrush with a precision (Compatible) or a power grip (Incompatible). A hand resting palm down on a table was used as baseline (Control). We hypothesized that if action simulation is causally involved in the affective response to art, subjects would like the artwork in the Compatible condition more than in the other two conditions.

## Materials and methods

### Participants

Twenty naïve healthy right-handed individuals (13 females; mean = 24 years) participated in the study. They were all naïve to the purpose of the investigation and with normal or corrected-to-normal vision.

### Stimuli

Stimuli consisted of 90 high quality color images of pointillist-style paintings (Table 1). Thirteen individuals (7 females; mean age = 27.9 years) who did not participate in the study pre-selected them among 200 canvases according to their style: pointillist-style, stroke-style, or otherwise. 90 images indicated as pointillist-style paintings by at least 10 out of 13 subjects were chosen for the experiment. Furthermore, three right gloved-hand images (holding a paintbrush with a power or a precision grip, or rested palm down) were used in the sensorimotor training (see Visuomotor Training) and as supraliminal priming images in the experiment (see Painting Observation and Liking Rates). All images were adjusted to the same size (470 × 351 pixels) using Adobe Photoshop and presented on a screen with a resolution of 1280 × 800 pixels, at 55 cm distance to subtend 12° horizontal and 9° vertical visual angles.

**Table 1:** **List of the pointillist-style paintings used in the experiment**.

**Surname**	**Name**	**Title**	**Surname**	**Name**	**Title**
Marevna		OбHaжeHHaя	Signac	Paul	Pine Tree at Saint-Tropez
Cross	Henri-Edmond	A Venetian Canal	Matisse	Henri	Le Cap Layet
Franco	Angelo	Blooming Tree	Matisse	Henri	Luxe, Calme et Volupté
Ferrigno	Andrea	Divide and Conquer	Matisse	Henri	Still Life
Franco	Angelo	Abstract Forest IV	Matisse	Henri	Still Life with Purro II
Zeniuk	Jerry	Untitled	Metzinger	Jean	Bathers, Two Nudes in an Exotic Landscape
Dellavallée	Henri	Farmyard	Metzinger	Jean	Bord de Mer
Dellavallée	Henri	La Rue au Soleil à Port-Manech	Metzinger	Jean	Femme Assise au Bouquet de Feuilage
Holton	William	Garden	Metzinger	Jean	Le Château de Clisson
Holton	William	Attractor	Metzinger	Jean	Nature Morte
Franco	Angelo	Forest Abstraction	Metzinger	Jean	Paysage au Deux Cypres
Franco	Angelo	Forest Abstraction #6	Metzinger	Jean	Paysage Neo-Impressiste
Franco	Angelo	Forest of Love	Metzinger	Jean	Matin au Parc Montsouris
Holton	William	Indra	Metzinger	Jean	Parc Monceau
Franco	Angelo	Virginia Forest Abstraction 1	Klee	Paul	Croix et Colonnes
Franco	Angelo	Floral Abstraction Verdant	Picabia	Francis	View of St. Tropez from the Citadel
Franco	Angelo	Manhattan Pidgeon	Picasso	Pablo	Le Retour du Bapteme, d'apres le Nain
Franco	Angelo	November Bouquet	Pissarro	Camille	Children on a Farm
Franco	Angelo	Nude Abstraction	Signac	Paul	Palais des Papes Avignon
Franco	Angelo	Portrait of a Hill	Franco	Sean	Bouquet in Ochre
Franco	Angelo	Rare Bird	Segal	Arthur	Marseille
Angrand	Charles	In the Garden	Seurat	Georges	The Maria—Honfleur
Angrand	Charles	Couple dans la Rue	Signac	Paul	The Port of Saint-Tropez
Balla	Giacomo	Girl Running on a Balcony	Signac	Paul	River's Edge—the Seine at Herblay
Cross	Henri-Edmond	The Golden Isles	Seurat	Georges	Port-en-Bessin—Avant-Port Marée Haute
Holton	William	Fallout	Seurat	Georges	Port-en-Bessin—Entrance to the Harbor
Cross	Henri-Edmond	Sunset on the Lagoon Venice	Signac	Paul	Les Andelys—the Riverbank
Signac	Paul	Saint-Tropez—the Storm	Seurat	Georges	Gravelines Annonciade
Cross	Henri-Edmond	Undergrowth	Lemmen	Georges	Factories on the Thames
Cross	Henri-Edmond	La Chaine des Maures	Goldstein	Leonard	Going Home in Black and White #1
Cross	Henri-Edmond	The Scarab	van Rysselberghe	Théo	Pointe Saint-Pierre at Saint-Tropez
Cross	Henri-Edmond	The Wood	Goldstein	Leonard	Shield of Moie
Cross	Henri-Edmond	Cypresses at Cagnes	Goldstein	Leonard	Flower Nebular #2
Dali	Salvador	Madrid, Architecture and Poplars	Luce	Maximilien	The Seine at Herblay
Dali	Salvador	Dawn, Noon, Sunset and Dusk	Luce	Maximilien	Montmartre—de la Rue Cortot, Vue vers Saint-Denis
Dali	Salvador	Bathers of Llane	Luce	Maximilien	Morning Interior
Derain	André	Boats at Collioure	van Rysselberghe	Théo	Sailboats and Estuary
Dubois	Louis	La Marne à l'Aube	Malevich	Kazimir	Landscape
Biggi	Gastone	Apalachi	van Dongen	Kees	Le Moulin de la Galette
Signac	Paul	Saint-Tropez—the Storm	Marevna		Flower Still Life
Biggi	Gastone	Odessa Chant	Kusama	Yayoi	Sunlight
Signac	Paul	View of Saint-Tropez	Lacombe	Georges	In the Forest
Vuillard	Edouard	My Grandmother	Lemmen	Georges	Beach at Heist
Biggi	Gastone	Attraversamenti	Lemmen	Georges	Heyst No.3 High Tide
Hofmann	Hans	Self Portrait	Lemmen	Georges	View of the Thames

### Visuomotor training

**Figure 1:**
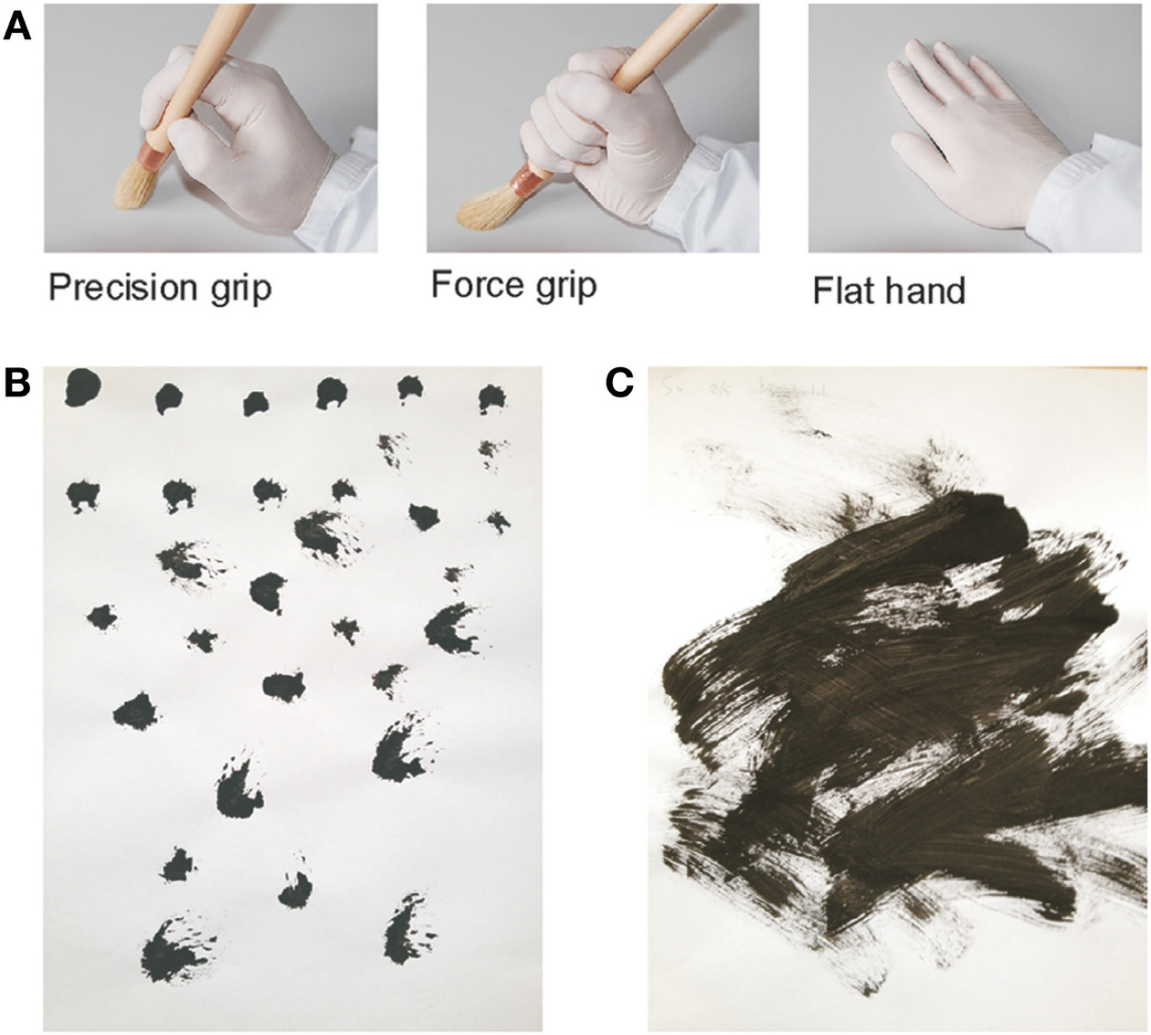
**Visuomotor training**. During the associative training, three images **(A)** depicting a right gloved-hand holding a paintbrush with a precision or a power grip (or rested palm down as control) instructed the participants to produce pointillist-style **(B)**, and stroke-style **(C)**, respectively.

We first established an association between the participants' own movements and the creation of pointillist-style or stroke-style paintings. To achieve this, we presented the participants with one out of three right gloved-hand images (Figure 1A) displayed on a screen (in random order, for 10 s, 6 times each) that served as instruction for the subjects to perform the desired training with the right hand.

The image of the hand holding a paintbrush with a precision grip instructed the participants to paint dots by executing stippling movements while holding the paintbrush with the precision grip (Figure 1B). The image depicting the hand holding a paintbrush with a power grip instructed the participants to paint strokes of about 10 cm by holding the paintbrush with a power grip (Figure 1C). The image depicting the hand rested palm down instructed the participants to position their hand palm down on the table. Task completion was supervised by the experimenter. The training was repeated before the first, third and sixth primed blocks (see below) for each grip (10 s each) to strengthen the visuomotor association.

### Painting observation and liking rates

**Figure 2:**
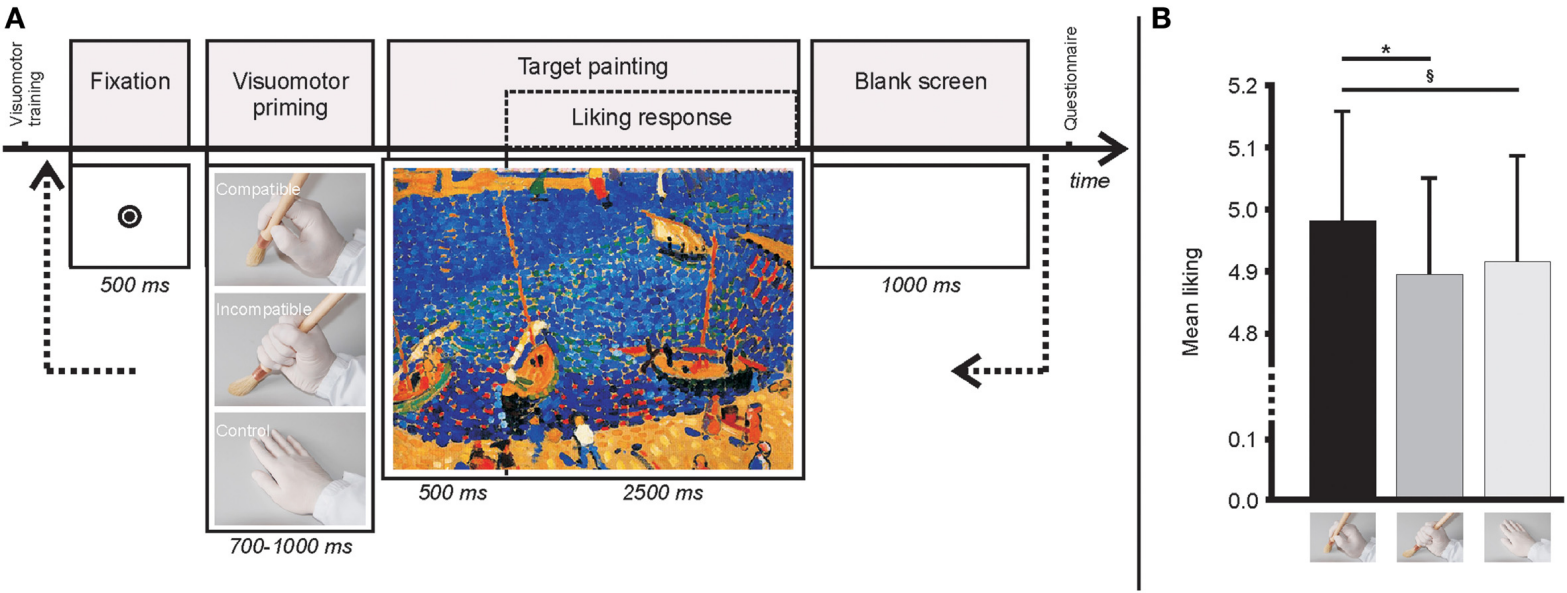
**(A)** Images of a gloved-hand holding a paintbrush were used as supraliminal priming before the display of each pointillist-style painting. The images consisted of either a precision or a power grip, or of a rested palm down hand and they created three conditions. *Compatible* (precision grip) or *Incompatible* (power grip) with the drawing of pointillist-style paintings. The palm down image served as *Control*. **(B)** The preference expressed when the paintings were preceded by priming images activating motor programs Compatible with the production of pointillist-style brushstrokes was higher than that expressed for the Incompatible (^*^p < 0.05) and the Control (marginally significant ^§^*p* = 0.067) conditions. The liking ratings in the Incompatible and Control conditions did not differ from each other (*p* = 0.567). Mean liking ratings in the three conditions are depicted (error bars represent s.e.m.).

After the visuomotor training, participants observed the 90 pointillist-style paintings preceded by one of the three images (700–1000 ms, randomly presented) depicting a right gloved-hand holding a paintbrush with a grip that supraliminally primed actions (for studies investigating how hand images prime actions see Borghi et al., 2007) that were either *Compatible* (precision grip) or *Incompatible* (power grip) with the drawing of pointillist-style paintings (Figure 2A). A palm down image served as *Control*. Each painting was presented three times, in nine randomized blocks (of 30 trials each) preceded by a different priming image. After 500 ms, the participants rated the paintings by moving a dot along a 9-point Likert-type scale displayed below the painting for 2500 ms (from “I like it very much” to “I do not like it at all,” direction counterbalanced across subjects) by left ring and index finger key-presses. Choices were confirmed by middle finger key-presses. A 1000 ms blank screen completed each trial. Due to the numerous unconfirmed ratings (≥10%) two participants were excluded from further analysis. In the remaining 18, a total of 3.25% of unconfirmed ratings was excluded.

### Familiarity

Upon completion of the experiment, the participants were debriefed to assess their familiarity with art by using an art questionnaire adapted from Chatterjee et al. (2010) by excluding questions 1–3 due to differences between the France and USA education systems. A median split (median of the Sums = 5.5) of the questions in Table 2 separated the participants into art-familiar and art-unfamiliar groups composed of nine participants each.

**Table 2:** **Art familiarity questionnaire**.

**Subject**	**Q1**	**Q2**	**Q3**	**Q4**	**Q5**	**Sum**	**Group**
1	2	2	0	0	1	5	1
2	4	4	0	0	0	8	2
3	2	2	0	0	0	4	1
4	2	2	0	0	0	4	1
5	4	2	0	0	0	6	2
6	4	4	0	0	0	8	2
7	4	2	0	0	2	8	2
8	5	3	0	2	2	12	2
9	5	4	2	1	5	17	2
10	2	1	0	0	1	4	1
11	2	0	0	0	0	2	1
12	0	0	0	0	0	0	1
13	2	0	0	0	0	2	1
14	0	0	0	0	0	0	1
15	4	4	0	6	2	16	2
16	3	2	0	1	2	8	2
17	2	1	0	1	1	5	1
18	4	4	0	1	1	10	2

Participants were divided into two Groups according to a questionnaire on art familiarity. Group 1 (median of the Sum < 5.5) and Group 2 (median of the Sum > 5.5) were composed of participants (nine in each group) with less or more art familiarity, respectively. Questionnaire: Q1. On average, you visit art museums about once every … (^*^); Q2. On average, you visit art galleries about once every… (^*^); Q3. In the average week how many hours do you spend making visual art? (range: “0” to “6 or above”); Q4. In the average week how many hours do you spend reading a publication that is related to visual art? (range: “0” to “6 or above”); Q5. In the average week how many hours do you spend each week looking at visual art? ^*^range for Q1 and Q2:“0” to “5.” 0 (almost never), 1 (once a year), 2 (once every 6 months), 3 (once every 2 months), 4 (once a month), 5 (once a week).

### Statistical analysis

To asses whether covert painting simulation modulated the liking rating, we entered the ratings in a 3 (Condition: Compatible, Incompatible, Control; within subjects) × 2 (Group: art-familiar, art-unfamiliar; between subjects) ANOVA. A significance threshold of *p* < 0.05 was set for all statistical tests.

## Results

The main factor Group [*F*_(1,16)_ = 0.665, *p* = 0.427, η^2^_*p*_ = 0.040] and the interaction Group × Condition [*F*_(2, 32)_ = 2.577, *p* = 0.092, η^2^_*p*_ = 0.139] were not significant. In other words, this result indicates that art familiarity did not influence the results. Instead, the factor Condition was significant [*F*_(2, 32)_ = 3.355, *p* = 0.047, η^2^_*p*_ = 0.173]. In particular, the aesthetic preference expressed for the paintings in the Compatible condition (4.974 ± 0.181; Mean ± s.e.m.) was significantly higher (*p* = 0.048, Newman-Keuls *post-hoc* test) than that in the Incompatible condition (4.877 ± 0.168), and marginally different (*p* = 0.067) from that in the Control condition (4.899 ± 0.176; Figure 2B). Instead, the liking rates did not differ between the Incompatible and Control conditions (*p* = 0.567).

Correlations between each condition and individuals' sum of experience ratings (see Table 2) were not significant (Pearson correlations *rs* < 0.236, *ps* > 0.346) thus ruling out any association between familiarity and liking scores.

## Discussion

In this behavioral study we show that the aesthetic appreciation for pointillist-style paintings is enhanced by presenting supraliminal action priming images that are congruent (Compatible condition) with the style required to create those paintings. How can the priming modulate liking ratings of passively observed canvases? We believe that the congruent priming facilitated the covert simulation of the brushstrokes present in the paintings, thus yielding to higher ratings. This interpretation is consistent with the hypothesis that motor structures have a role in aesthetic and particularly that involuntary painting simulation contributes to aesthetic appreciation (Freedberg and Gallese, 2007; Leder et al., 2012; Umiltà et al., 2012). In agreement with previous work (Umiltà et al., 2012), our results also suggest that this effect is independent of familiarity with art. Nonetheless, since all participants were not actively engaged in creating artwork (see Question 3 in the Art familiarity questionnaire, Table 2) we cannot rule out the possibility that the results would be different for artists.

What is the mechanisms involved in simulating brushstrokes? The concept of covert action simulation has acquired a new interest with the work conducted on the mirror neuron mechanism in the non-human and human primate brain (Rizzolatti and Sinigaglia, 2010). Through this mechanism, other agents' actions are mirrored in one's own motor system thus, it is thought, helping to understand others' motor acts from “within.” Action of other agents can be mirrored or covertly simulated when they are directly observed as well as when they are represented as static pictures (i.e., images depicting body movements, see Mado-Proverbio et al., 2009; Urgesi et al., 2010), and when they are hidden from view and only their sound (Ticini et al., 2012) or their traces (Longcamp et al., 2003) are perceived. For instance, there is evidence that observation of hand written letters triggers activity in motor areas involved in writings (Longcamp et al., 2003; see also Ticini, 2013), and particularly that learning to write facilitates the visual recognition of letters through the participation of brain areas known to be activated by the execution, imagery and observation of actions (Longcamp et al., 2008). Our result is supported by these and more recent behavioral findings reporting that the direction of observed brushstrokes affects participants' response speed in reaction time experiments (Taylor et al., 2012) and that *active* execution of movements increases (or decreases) the viewer's liking ratings when they match (or not) the style of the painting (Leder et al., 2012).

These results could be also explained by alternative mechanisms not necessarily involving painting simulation. For instance, it is plausible that the implicit knowledge about the correct action needed to manipulate the paintbrush (see Buxbaum and Kalenine, 2010) may have facilitated the most functional and effortless motor program to grasp a brush in order to create pointillist-like paintings. This would be in accordance with the idea that fluency in stimulus processing can influence aesthetic responses, as well (Reber et al., 2004). Moreover, unlike in Leder et al. (2012), we cannot exclude that self-observation of one own's hands during the training may have strengthened visuo-visual (instead of visuo-motor) associations between the hand grip and the painting style. We also cannot exclude that an intrinsic affective value of the action primes may have biased the preference ratings (e.g., the precision grip could have been perceived as more positive than the power grip). In this regard, a recent article from Flexas et al. showed differences in liking for abstract artwork when they were preceded by facial primes showing happiness, disgust or no emotion (Flexas et al., 2013). In particular, paintings preceded by happiness primes were liked more than those preceded by disgust primes. If it were the case in our experimental setup, our results would extend previous research on how the affective transfer elicited by priming may influence evaluative judgments (e.g., Murphy and Zajonc, 1993; Rotteveel et al., 2001) to the domain of aesthetic experience. Finally, we cannot exclude that the prior training alone could be sufficient to enhance the ratings as a result of an exposure effect, without the need of priming images presented before each painting.

In conclusion, we here provide empirical evidence that, beyond other factors such as upbringing, historical context and nature of the artistic stimuli, covert painting simulation may influence affective responses to art (Freedberg and Gallese, 2007). Although we cannot fully rule out alternative explanations, we suggest that the contribution of motor areas may be fundamental for the attribution of the hedonic value to some objects of art. Since simulation appears pivotal for understanding the actions and emotions of others, one important area of future research will be to characterize its influence on affective centers beyond the domain of artistic preference. Obtaining a better understanding of the contribution of action simulation in affective states is likely to shed light not just on how the brain encodes affective stimuli but also may enrich our perspective on the neural mechanisms involved in some social and communicative deficits associated with action simulation, such as autism spectrum disorder (Oberman and Ramachandran, 2007).

### Conflict of interest statement

The authors declare that the research was conducted in the absence of any commercial or financial relationships that could be construed as a potential conflict of interest.
